# Toddlers Selectively Help Fair Agents

**DOI:** 10.3389/fpsyg.2017.00944

**Published:** 2017-06-07

**Authors:** Luca Surian, Laura Franchin

**Affiliations:** Department of Psychology and Cognitive Sciences, University of TrentoRovereto, Italy

**Keywords:** selective helping, fairness, reciprocity, infant, social cognition, distributive justice

## Abstract

Previous research showed that infants and toddlers are inclined to help prosocial agents and assign a positive valence to fair distributions. Also, they expect that positive and negative actions directed toward distributors will conform to reciprocity principles. This study investigates whether toddlers are selective in helping others, as a function of others’ previous distributive actions. Toddlers were presented with real-life events in which two actresses distributed resources either equally or unequally between two puppets. Then, they played together with a ball that accidentally fell to the ground and asked participants to help them to retrieve it. Participants preferred to help the actress who performed equal distributions. This finding suggests that by the second year children’s prosocial actions are modulated by their emerging sense of fairness.

**Highlights:**

Toddlers (mean age = 25 months) are selective in helping distributors.

Toddlers prefer helping a fair rather than an unfair distributor.

Toddlers’ selective helping provides evidence for an early sense of fairness.

## Introduction

Cooperation, the acting together for a common end or purpose (Tuomela, 1993), is an essential aspect of human life, it is necessary to its survival as well as to its flourishing. In order to work, it requires good skills in partner choice, allowing the individual to stay away from free-riders, non-reciprocators, and other unfair and harmful individuals (Fehr et al., 2008; Baumard et al., 2013; Shaw et al., 2014). Cooperation also requires an understanding of others’ goals and it is grounded in social motivations for helping and sharing with others (Tomasello, 2007). What are the origins of these motivations and evaluation skills? Recent works on infants’ social preferences and expectations help us to address this question and constraint nativist and empiricist models of cognitive architecture and development (Margolis and Laurence, 2013; Tomasello and Vaish, 2013). In the first year, infants prefer agents who help others to agents who hinder others (Hamlin et al., 2007, 2011; Hamlin, 2013, 2015) and agents who comfort rather than harm (Buon et al., 2014; see also Holvoet et al., 2016). By 10 months, they expect agents to perform equal distributions (Meristo et al., 2016) and to act positively toward fair donors and negatively toward unfair donors (Meristo and Surian, 2013, 2014).

In their second year, infants also evaluate distributions of resources performed toward third parties (Schmidt and Sommerville, 2011; Sommerville et al., 2013), choose agents who perform equal rather than unequal distributions (Geraci and Surian, 2011; Burns and Sommerville, 2014) and take relative merit into account when witnessing equal and unequal distributions (Sloane et al., 2012). Also, they associate praise and admonishments to fair and unfair distributors, respectively (DesChamps et al., 2015). Overall, this evidence suggests that they assign a positive valence to fair distributions and expect that positive and negative actions directed toward distributors will conform to reciprocity principles (Baillargeon et al., 2015).

Most of the available evidence on infants’ emerging ability to evaluate distributions comes from looking times. In the present study we looked for converging evidence from a more active dependent measure, namely infants’ prosocial actions performed toward agents that have just distributed resources fairly or unfairly. Prosociality may be expressed through a variety of activities, such as giving instrumental help and sharing resources, which can be observed from early childhood. Toddlers display spontaneous helping when see an adult having trouble achieving a goal (e.g., Warneken and Tomasello, 2006, 2007) and they display sharing actions when they recognize another individual’s lack of a desired material good (Olson and Spelke, 2008; Svetlova et al., 2010; Dunfield et al., 2011).

One crucial aspect of early prosocial actions is their selectivity (Kuhlmeier et al., 2014). Dunfield and Kuhlmeier (2010) found that 21-month-olds selectively picked up an out-of-reach object for an actor who, in a previous interaction, intended to provide them with a desired toy over one who did not. Toddlers selected the recipient of their helping behavior based on the helpee’s intention to deliver toy, even if he or she failed to deliver it. Likewise, 3-year-olds selectively avoid helping those who caused harm, or even simply intended to cause harm to others (Vaish et al., 2010). Previous findings also indicate that toddlers and young children select the recipient of their sharing behaviors relying on recipients’ previous actions (Olson and Spelke, 2008; Kenward and Dahl, 2011; Kanngiesser and Warneken, 2012).

In this study, we examined whether selective helping in toddlers is linked to previous distributive actions. We recorded their helping behaviors toward agents that performed either equal or unequal allocations of resources. Previous results on toddlers’ propensity to help prosocial agents and evaluate distributors lead to the prediction that they would be more likely to help a fair than an unfair distributor.

## Materials and Methods

### Participants

Participants were 44 healthy and full-term toddlers (22 girls; mean age = 25 months, 18 days; age range: 18 months, 12 days to 33 months, 6 days). All participants were recruited at public and private nurseries of two northern Italian cities. Five additional toddlers were excluded from analysis as a result of experimenter error. Children were tested only after obtaining their parents’ written informed consent. The research project was approved by the University of Trento Ethical Committee.

### Materials and Procedure

**FIGURE 1:**
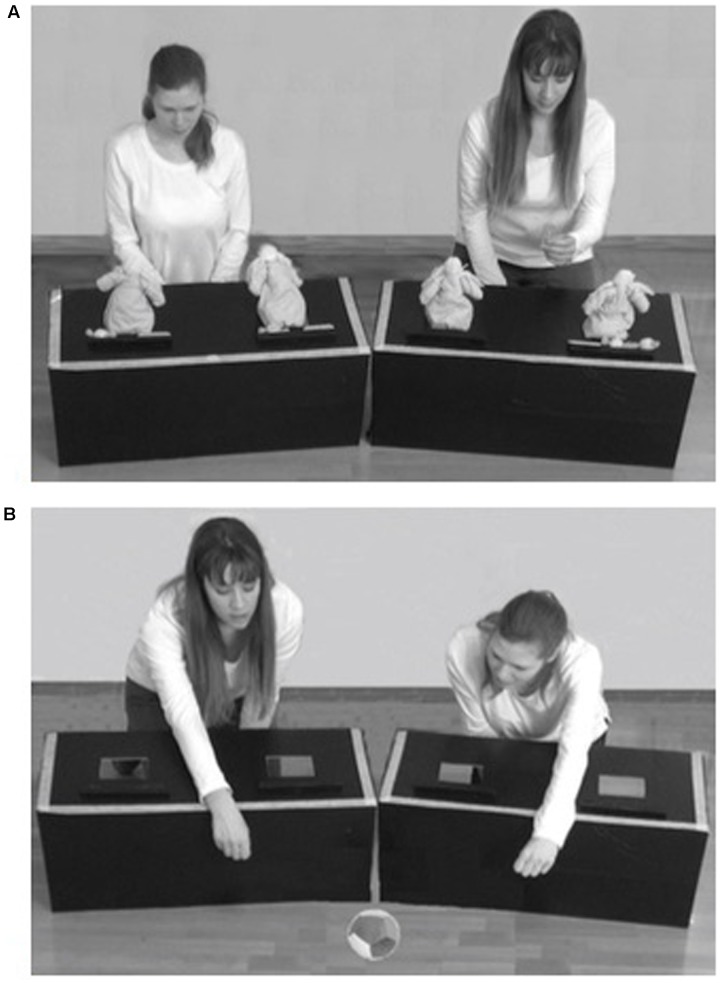
Illustrations of the distribution and test phases in the selective helping task. During the distribution phase **(A)**, the two actresses distributed biscuits and candies. One of them performed two equal distributions toward her two puppets while the other one performed two unequal distributions by giving everything to one of her two puppets. During the test phase **(B)**, the actresses first played with a ball and then asked toddlers to help them to retrieve the out of reach ball.

Children were tested in a quiet room of the nurseries. They sat on an educator’s lap on the floor, centered in front of two black boxes (33 cm × 77.5 cm × 31 cm) at a distance of 80 cm (see **Figure 1**).

Following Dunfield and Kuhlmeier (2010), all testing sessions started with a familiarization phase during which children were introduced by the experimenter to two couples of identical puppets (‘sisters’). After placing all the puppets into four slots (two in each box, see **Figure 1**), the experimenter played with a sand-ball that accidentally fell to the floor and she said: “Oh no! Help me! I dropped the ball, can you help me?” The experimenter waited for a child’s response, outstretching her hand toward him/her. When the child gave the ball to the experimenter, she/he was presented with real-life events in which two actresses divided resources (i.e., cookies and candies) between the two couples of puppets. The actresses were presented as the mothers of the two puppets who came to play with them. One actress, the fair one, equally distributed her resources between the puppets by saying: “I have two cookies, one for you and the other for you.” The other actress, the unfair one, unequally allocated all her resources to one of the puppets and gave nothing to the other one. However, she interacted verbally with both puppets by saying: “I have two cookies, one for you, the other one again for you (looking at one of the two puppets), and for you (looking at the other puppet): nothing.” In total, children were presented with four distributions: two of cookies and two of candies. One actress allocated always equally her cookies and candies, while the other actress distributed always unequally her resources.

Then, after hiding the puppets and the resources, the actresses facing each other played together with a sand-ball. After four throws, they placed the ball between the two boxes and then the ball ‘accidentally’ fell to the ground. The actresses said simultaneously and only once: “On no! Help us!,” and waited for a child’s response, outstretching their hand toward the ball, maintaining neutral expressions and avoiding eye contact with the participant. The child had to choose which actress to help. The test trial ended after a choice was made or after 90 s elapsed without a choice.

We counterbalanced across participants the following factors: side of the fair Helpee, side of first distributive action, order of the distributions (fair first vs. unfair first), and identity of the (fair and unfair) Helpee. The two actresses had similar clothes and behaved similarly, except for their distributive behavior, and maintained a neutral facial expression during the entire test.

Coding children’ choices of Helpee was carried out by examining the video recordings. Two coders carried out this evaluation, one of them was ignorant about the actresses’ fair and unfair actions. There was 100% agreement between coders.

### Statistical Methods

Since the aim of the study was to compare the percentage of binary choices of children as well as to test the hypothesis that the overall percentage of choices of children for the fair actress was different from 50%, the Fisher’s exact test and the exact binomial test were employed (two-tailed). Binomial tests were also suitable to analyze the effects of side of the fair actress, side of first object-giving action at the start of the distributions, order of the fair and unfair distributions, and actress’ identity.

## Results

None of the counterbalanced factors had a significant effect on children’s choices (*p*’s > 0.07). Children showed no uncertainty in their choices by starting to give the ball to one actress, but finally giving it to the other one. Eleven children (25%) did not help either actress and the rest (*n* = 33, 75%) helped one of them. This helping rate is similar to the rate found in previous experiments (Dunfield and Kuhlmeier, 2010). Twenty-four children (72.7%) helped the fair actress and the other 9 (27.3%) helped the unfair one, binomial test, *p* = 0.014.

Considering the wide age range of the participants, we split the sample in two age groups: 22 20-month-olds (11 girls; mean age = 20 months, 29 days; age range: 18 months, 12 days to 23 months, 4 days) and 22 30-month-olds (11 girls; mean age = 30 months, 8 days; age range: 27 months, 17 days to 33 months, 6 days). The preference for the fair actress in the two age groups was very similar, Fisher exact test, *p* > 0.5, *OR* = 1.24 95% CI [0.21, 7.94]. At 20 months, 17 children (77.3%) helped one of the actresses. Twelve children (71%) helped the fair actress, and the other 5 (29%) helped the unfair actress. At 30 months, 16 children (72.7%) helped one of the actresses, 12 (75%) helped the fair actress and 4 (25%) the unfair one. Due to the small sample size, the *p*-values of the separate binomial tests were above the 0.05 significance level and reached trend level in the older group (*p* = 0.142 and *p* = 0.076, respectively).

## Discussion

These results reveal that toddlers’ selective helping is linked to agents’ previous distributive actions. This finding contributes to our understanding of early social cognition in two important ways. First, it shows that selective helping is not constrained solely by agents’ helping or hindering actions, but also by their distributive behaviors previously directed toward third parties. Second, it points out that toddlers’ sensitivity to agents’ distributive actions may inform processes underpinning their prosocial choices, thus providing crucial converging evidence to support recent proposals on the early emergence of a sense of fairness.

Preferences for prosocial agents have been reported in several experiments, even in preverbal infants, using scenarios in which helpers facilitate a third party in achieving a goal while hinderers prevent the goal achievement (e.g., Hamlin et al., 2007, 2011). However, a preference for fair distributors has been reported in only two previous studies. This preference was found at 15–16 months, but not before (Geraci and Surian, 2011; Burns and Sommerville, 2014). Therefore, the present study provides an important contribution to the literature on infants’ sense of fairness by consolidating and extending the effects found with paradigms that require an action response that reveals a preference for a certain type of distributors.

One limitation of the present study derives from the relatively small sample size. An interesting goal for future studies on infants’ and toddlers’ selective helping will be to employ longitudinal designs and to assess whether their preference for fair individuals develops simultaneously or later than their preference for agents that comfort (Buon et al., 2014) and help others (Hamlin, 2013). Future works could extend this result in several interesting ways. By presenting equal and unequal distributors paired with neutral agents one could test whether the bias found in the present study was due to a preference for helping the fair agent or to avoid the unfair one (Hamlin et al., 2007; Vaish et al., 2010), or both. It would be also useful to test children in a non-distributive control condition, like in Meristo et al. (2016, Exp. 3), in order to assess the possible effect due to the symmetric and asymmetric interactions displayed by the two agents.

Moreover, one way to cast light on the underlying mechanism would be to investigate whether distributors’ intentions to perform equal or unequal distributions can affect toddler’s selective helping, regardless of the final outcome of their attempts (Margoni and Surian, 2016). This would also help to assess the robustness and generality of toddlers’ intention-mediated selecting helping, previously found (Dunfield and Kuhlmeier, 2010) in harming and helping scenarios.

## Author Contributions

Both authors contributed equally to hypothesis generation, experimental design, data collection and analyses, and writing of the manuscript.

## Conflict of Interest Statement

The authors declare that the research was conducted in the absence of any commercial or financial relationships that could be construed as a potential conflict of interest. The reviewer AP and handling Editor declared their shared affiliation, and the handling Editor states that the process nevertheless met the standards of a fair and objective review.
